# Increased Remnant Lipoproteins in Apo E Deficient Mice Induce Coronary Atherosclerosis following Transverse Aortic Constriction and Aggravate the Development of Pressure Overload-Induced Cardiac Hypertrophy and Heart Failure

**DOI:** 10.3390/biomedicines10071592

**Published:** 2022-07-04

**Authors:** Ilayaraja Muthuramu, Mudit Mishra, Bart De Geest

**Affiliations:** 1 Centre for Molecular and Vascular Biology, Catholic University of Leuven, 3000 Leuven, Belgium; ilaya@upenn.edu (I.M.); drmuditm@gmail.com (M.M.); 2 Gene Therapy Program, Department of Medicine, Perelman School of Medicine, University of Pennsylvania, Philadelphia, PA 19104, USA; 3 Department of Cardiothoracic Surgery, University Medical Center Utrecht, 3508 GA Utrecht, The Netherlands; 4 Laboratory of Experimental Cardiology, Department of Cardiology, University Medical Center Utrecht, University Utrecht, 3508 GA Utrecht, The Netherlands

**Keywords:** coronary atherosclerosis, pathological hypertrophy, heart failure, apolipoprotein E, remnant lipoproteins, oxidative stress, cardiac dysfunction, pressure overload, transverse aortic constriction

## Abstract

Murine coronary arteries are very resistant to the development of atherosclerosis, which may be related to their intramyocardial course. Blood pressure promotes atherosclerotic plaque formation by acting as a physical force that potentiates the migration of pro-atherogenic lipoproteins across the endothelium. C57BL/6N apolipoprotein (apo) E deficient mice have increased remnant lipoproteins that are a risk factor for coronary atherosclerosis. In this study, our aim was to quantify coronary atherosclerosis and artery remodeling following transverse aortic constriction (TAC) in C57BL/6N apo E^−/−^ mice and to evaluate the impact of increased remnant lipoproteins on the development of pressure overload-induced cardiac hypertrophy and heart failure. Advanced atherosclerotic lesions were observed in the left coronary artery of C57BL/6N apo E^−/−^ TAC mice but not in C57BL/6N TAC mice. Pressure overload resulted in markedly increased cardiac hypertrophy and more pronounced heart failure in C57BL/6N apo E^−/−^ TAC mice in comparison to C57BL/6N TAC mice. Pathological hypertrophy, as evidenced by increased myocardial fibrosis and capillary rarefaction, was more prominent in C57BL/6N TAC apo E^−/−^ than in C57BL/6N TAC mice and led to more marked cardiac dysfunction. In conclusion, TAC in apo E deficient mice induces coronary atherosclerosis and aggravates the development of pathological cardiac hypertrophy and heart failure.

## 1. Introduction

All pro-atherogenic lipoproteins contain apolipoprotein (apo) B. Retention of pro-atherogenic lipoproteins within the vessel wall is the obligatory initiating event of atherogenesis [1,2]. The retained lipoproteins trigger an inflammatory response that is accompanied by lesion progression and the formation of advanced atherosclerotic plaques [2]. Apo B-containing lipoproteins stick to the vessel wall mainly through electrostatic binding to proteoglycans in the extracellular matrix. Ionic binding occurs between positively charged amino acids in apo B-100 and the negatively charged glycosaminoglycans [3]. Furthermore, there is a distinct functional proteoglycan-binding site on apo B-48, and apo B-48-containing lipoproteins bind proteoglycans with similar affinity compared to apo B-100-containing lipoproteins [4]. Areas prone to developing atherosclerotic lesions, such as branch sites, often contained distinct amounts and types of glycosaminoglycans [5]. In addition, glycosaminoglycan composition differs between distinct vascular beds within one species and within the same vascular bed between different species [5]. These observations may partially explain the different susceptibility of different arteries to atherosclerosis within the same species or differences in atherosclerosis susceptibility of the same artery between different species.

Distinct cardiovascular risk factors may have a differential effect on the endothelial cells and vascular smooth muscle cells at distinctive parts of the vascular tree [6]. Observational data from the Pathobiological Determinants of Atherosclerosis in Youth study indicate that the effect of cardiovascular risk factors on atherogenesis is dependent on the specific vascular bed [7,8,9]. Type III hyperlipoproteinemia, characterized by the presence of large amounts of chylomicron remnants and very low-density lipoprotein (VLDL) remnants, not only induces coronary atherosclerosis but also frequently leads to peripheral vascular disease and cerebral vascular disease [10,11,12]. In contrast, type IIa hyperlipidemia in patients with familial hypercholesterolemia characterized by increased levels of low-density lipoprotein (LDL) cholesterol specifically induces atherosclerotic plaque deposition in the coronary arteries and proximal aorta and does not lead to atherosclerosis in other vascular beds when unescorted by additional cardiovascular risk factors [13]. The relative atherogenicity of VLDL and LDL has been demonstrated to differ substantially in different parts of the rabbit aorta in a direct comparison of two rabbit models with matched total cholesterol levels but distinct amounts of VLDL cholesterol and LDL cholesterol [14,15].

A particular observation is that the coronary arteries of mice are very resistant to the development of atherosclerosis [16]. Atherosclerotic plaques in humans predominantly develop in the coronary and carotid arteries, whereas in mice, lesions are mainly localized in the aortic sinus, the proximal aorta and the aortic arch, and the brachiocephalic trunk [16]. An apparent exception to this rule is the fact that mice with homozygous null mutations in the genes for both scavenger receptor class B, type I (SR-BI), and apo E develop severe occlusive, fibrin-containing coronary arterial lesions but all die by 8 weeks of age [17]. The time course and the specific pathological observations in double SR-BI apo E knockout mice indicate that these mice rather represent a model of a complex aggressive obliterative arteriopathy and not of atherosclerosis. The resistance of coronary arteries in mice to coronary atherosclerosis may be related to the proteoglycan composition of the subendothelium. However, one feature of coronary arteries in mice is the intramyocardial course. Intramyocardial arteries, in general, do not develop atherosclerosis [18]. This may be caused by the lower or absent transmural pressure gradient in intramyocardial arteries [18].

Patients with hypertension have increased presence, extent, and severity of coronary atherosclerosis quantified by coronary computed tomographic angiography [19]. Blood pressure promotes atherosclerotic plaque formation by acting as a physical force that potentiates the migration of pro-atherogenic lipoproteins across the endothelium. Hypertension is also a principal risk factor for the development of heart failure [20].

Since transverse aortic constriction (TAC) between the origin of the right innominate artery and the left common carotid artery induces a rise in blood pressure proximal to the constriction, this procedure raises coronary pressure. Notwithstanding this, coronary atherosclerosis is completely absent in several murine models of hypercholesterolemia or dyslipidemia following TAC [21,22,23]. Increased remnant lipoproteins are a risk factor for ischemic heart disease and myocardial infarction in humans [24,25]. We speculated that increased remnant lipoproteins in apolipoprotein (apo) E deficient mice might produce coronary atherosclerosis under conditions of increased coronary pressure. The specific objectives of this study were 1) to quantify coronary atherosclerosis and artery remodeling in C57BL/6N apo E^−/−^ TAC mice and 2) to evaluate the impact of increased remnant lipoproteins on the development of pressure overload-induced cardiac hypertrophy and heart failure.

## 2. Materials and Methods

### 2.1. In Vivo Experiments

All investigations were performed in accordance with the European legislation on protection of animals used for scientific purposes (Directive 2010/63/EU), and all experimental procedures on animals were performed in accordance with protocols approved by the Institutional Animal Care and Research Advisory Committee of the KU Leuven (Approval number: P154/2013). Mice were housed in filter top type II cages containing 5 mice or less in the semi-specific pathogen-free facility of KU Leuven at Gasthuisberg. A semi-natural 12 h cycle of light and dark was maintained, and temperature was kept in a range from 21 °C to 22 °C. C57BL/6N mice and C57BL/6N apo E^−/−^ mice, originally purchased from Taconic (Ry, Denmark), were locally bred in the animal unit at Gasthuisberg and were fed standard chow diet (Sniff Spezialdiäten GMBH, Soest, Germany). Apo E deficient mice from Taconic had been backcrossed to the C57BL/6N background for 11 generations and were backcrossed for one additional generation. All experiments were performed on female mice. In order to induce pressure overload, transverse aortic constriction (TAC) was performed at the age of 14 weeks, as described in detail before [26,27]. The position of the TAC was between the origin of the right innominate artery and the left common carotid artery. The surgical procedure in sham mice was identical, except that no constriction on the aorta was applied. In the first experimental layer, C57BL/6N mice and C57BL/6N apo E^−/−^ mice were assigned for hemodynamic quantification and morphometric and histological analyses 8 weeks following the sham or TAC operation. The second experimental layer consisted of mice that did not undergo perfusion fixation and that were used for unbiased quantification of tissue and organ weights 8 weeks after the TAC or sham procedure.

### 2.2. Blood Sampling

Blood was collected by puncture of the vena cava inferior at the end of the experiment just before euthanasia. Anticoagulation was performed with 0.1 volume of 136 mmol/L trisodium citrate. Subsequently, plasma was isolated by centrifugation at 1100× *g* for 10 min and immediately stored at −80 °C.

### 2.3. Separation of Lipoproteins by Gel Filtration

Murine lipoproteins were fractioned by fast performance liquid chromatography gel (FPLC) filtration (Waters Associates, Milford, MA, USA) of 100 µL plasma obtained from C57BL/6N mice or from C57BL/6N apo E^−/−^ mice as described previously [28]. Cholesterol levels in high-density lipoprotein (HDL) and non-HDL fractions were determined by Amplex^TM^ Red Kit (Molecular Probes, Carlsbad, CA, USA).

### 2.4. Quantification of Lipid Peroxidation Products in Plasma

Lipid peroxidation was analyzed by measurement of Thiobarbituric Acid Reactive Substances (TBARS). Quantification of TBARS was performed according to the instructions of the manufacturer (Cayman Chemical, Ann Arbor, MI, USA).

### 2.5. In Vivo Hemodynamic Measurements

Invasive hemodynamic measurements were executed 8 weeks after sham operation or TAC using a 1.0 French Millar pressure catheter (SPR-67/NR; Millar instruments, Houston, TX, USA) as described. In order to perform these measurements, anesthesia was induced by intraperitoneal administration of 1.2 g/kg urethane (Sigma, St. Louis, MO, USA). Hemodynamic data were registered with Powerlab Bridge Amplifier and Chart Software (sampling rate 2000 Hz; ADInstruments Ltd., Oxford, UK).

### 2.6. Histological Analysis

Histological analysis was performed as described before [29]. Mice that first underwent hemodynamic analyses were perfused via the abdominal aorta with phosphate-buffered saline. Subsequently, hearts were arrested in diastole by administration of KCl (100 μL; 0.1 mol/L), followed by perfusion fixation with 1% paraformaldehyde in phosphate-buffered saline. Post-fixation overnight was performed with 1% paraformaldehyde, and hearts were subsequently embedded in paraffin. Cross-sections of 6 μm thickness at 130 μm spaced intervals were made, extending from the apex to base of the heart.

Sirius Red staining was performed to quantify interstitial collagen content [30]. Sirius Red polarization microscopy on a Leica RBE microscope with KS300 software (Zeiss) was applied to quantify thick, tightly packed mature collagen fibers as orange-red birefringent and loosely packed less cross-linked and immature collagen fibers as yellow-green birefringent. Collagen positive area was normalized to the left ventricular remote area and was expressed as percentage. To quantify interstitial collagen, any perivascular fibrosis was excluded from the analysis. Perivascular fibrosis was determined as the ratio of the fibrosis area surrounding the vessel to the total vessel area. Capillary density in the myocardium was quantified on CD31-stained sections using rat anti-mouse CD31 antibodies (BD Biosciences, Erembodegem, Belgium; dilution 1/500). Two mid-ventricular cross-sections were analyzed per mouse [31,32].

Immunostaining for 3-nitrotyrosine with rabbit anti-nitrotyrosine antibodies (Merck Millipore, Overijse, Belgium; dilution 1/250) was applied to evaluate nitro-oxidative stress [21].

Morphometric analyses of the left coronary artery were performed on Sirius Red-stained sections at 130 μm spaced intervals.

### 2.7. Statistical Analysis

At the completion of the study, data of all surviving mice were included in the analysis. All investigators who performed different endpoint analyses were blinded to group allocation. Unblinding of animal numbers corresponding to specific allocation groups was carried out at completion of measurements. All data are expressed as means ± standard error of the means (SEM). Parameters between two groups (cholesterol data, hemodynamic parameters) were compared using Student’s *t*-test using GraphPad Instat (GraphPad Software, San Diego, CA, USA). When indicated, a logarithmic transformation, a square root transformation, or a non-parametric Mann–Whitney test was conducted. The assumption of Gaussian distribution was checked using the method Kolmogorov and Smirnov. Parameters between four groups were compared by one-way analysis of variance followed by Bonferroni multiple comparisons post-test for comparing the sham groups, the TAC groups, and the sham versus respective TAC groups using GraphPad Instat. When the assumption of sampling from populations with identical standard deviations was not met, a logarithmic transformation or a square root transformation was applied. When the assumption of sampling from populations with Gaussian distributions was not met, a Kruskal–Wallis test was executed, followed by Dunn’s multiple comparisons post-test. Kaplan–Meier survival curves were analyzed by log-rank test using Prism4 (GraphPad Software). A two-sided *p*-value of less than 0.05 was considered statistically significant.

## 3. Results

### 3.1. Markedly Increased Mortality in C57BL/6N Apo E^−/−^ Mice Compared to C57BL/6N Mice following Transverse Aortic Constriction

**Figure 1:**
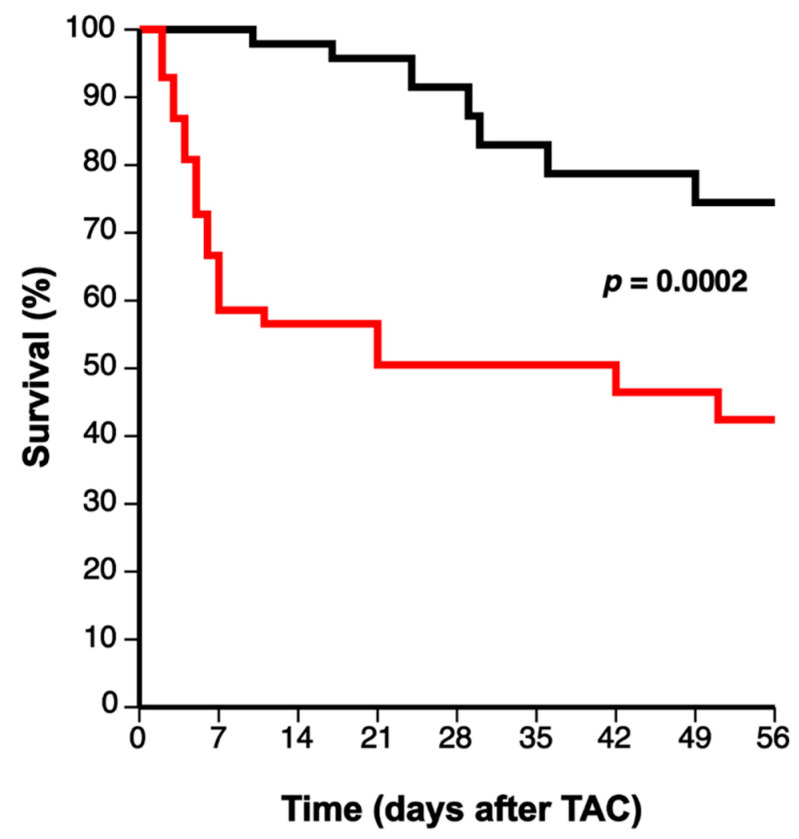
Comparison of Kaplan–Meier survival curves during 8 weeks follow-up after TAC. Female C57BL/6N TAC mice (black line) and female C57BL/6N apo E^−/−^ TAC mice (red line) are compared. The time-point of 0 days corresponds to the induction of TAC at the age of 14 weeks. Comparison of survival curves was performed by log-rank test.

Transverse aortic constriction (TAC) was performed at the age of 14 weeks to induce pressure overload. Comparison of Kaplan–Meier survival curves by log-rank test demonstrated a pronounced and highly statistically significant increased mortality rate in C57BL/6N apo E^−/−^ TAC mice compared to C57BL/6N TAC mice (hazard ratio for mortality 2.59, 95% confidence interval 1.56 to 4.30; *p* = 0.0002) during a follow-up period of 8 weeks (Figure 1). Eight weeks after surgical intervention, 35 out of 47 (74.5%) C57BL/6N TAC mice and 42 out of 99 (42.4%) C57BL/6N apo E^−/−^ TAC mice were alive (Figure 1). Sham operation in both C57BL/6N apo E^−/−^ and C57BL/6N mice did not result in any mortality (data not shown).

### 3.2. Pressure Overload Induced by TAC Induces Coronary Atherosclerosis in C57BL/6N Apo E^−/−^ Mice

**Figure 2:**
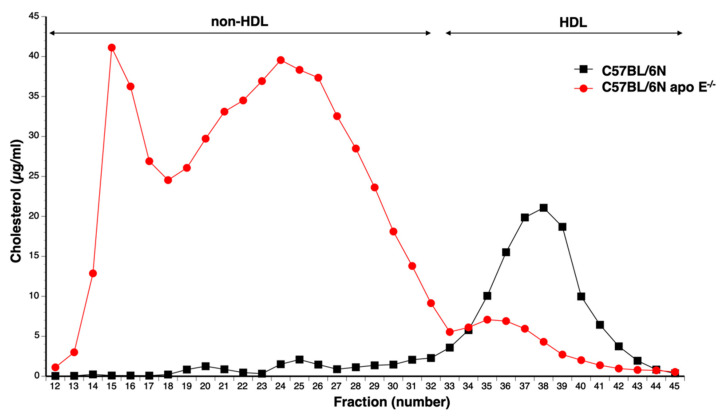
Separation of lipoproteins by gel filtration. Cholesterol lipoprotein profile in female C57BL/6N and female C57BL/6N apo E^−/−^ mice. Plasma samples were fractionated on a Superdex^®^ Gel Filtration Column (Sigma, St. Louis, MO, USA). Fractions 12 to 32 contain non-HDL whereas HDL is comprised in fraction 33 to 45.

Increased remnant lipoproteins and hypertension are two independent risk factors for coronary atherosclerosis. We speculated that both risk factors might interact to produce coronary atherosclerosis in C57BL/6N apo E^−/−^ mice. Since the genetic background may have a profound effect on cholesterol levels in apo E deficient mice [33], we first compared the lipoprotein profiles of apo E^−/−^ mice backcrossed for 12 generations to the C57BL/6N background and wild-type C57BL/6N mice (Figure 2). Total plasma cholesterol levels were 4.33-fold (*p* < 0.0001) higher in C57BL/6N apo E^−/−^ mice than in C57BL/6N mice (Figure 2, Table 1). This increase was entirely attributable to an increase in non-HDL cholesterol levels, whereas HDL cholesterol levels in C57BL/6N apo E^−/−^ mice were 61.8% (*p* < 0.0001) lower than in C57BL/6N mice (Figure 2, Table 1).

**Figure 3:**
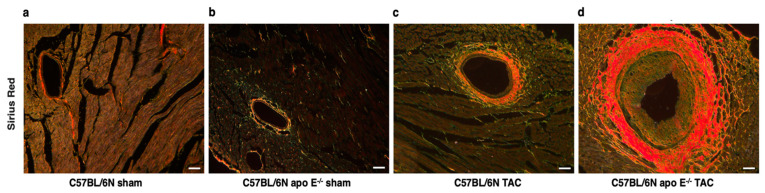
Left coronary artery sections illustrating coronary artery remodeling and coronary atherosclerosis. Representative Sirius Red-stained sections of the left coronary artery of female C57BL/6N sham mice (**a**), female C57BL/6N apo E^−/−^ sham mice (**b**), female C57BL/6N TAC mice (**c**), and female C57BL/6N apo E^−/−^ TAC mice (**d**). The media is demarcated by the internal elastic lamina (IEL) and the external elastic lamina (EEL). Atherosclerosis is present in intimal tissue within the IEL. The scale bar represents 50 µm.

Data on coronary artery remodeling and coronary atherosclerosis are provided in Table 2. Representative Sirius Red-stained cross-sections of left coronary arteries are shown in Figure 3. Pressure overload in C57BL/6N mice resulted in outward remodeling, as evidenced by a 2.19-fold (*p* < 0.001) increase in the area within the external elastic lamina. In addition, pressure overload in C57BL/6N TAC mice resulted in media hypertrophy, as evidenced by a 1.73-fold (*p* < 0.001) increase in the medial area compared to C57BL/6N sham mice. The luminal area was 2.50-fold (*p* < 0.001) higher in C57BL/6N TAC mice than in C57BL/6N sham mice. These changes reflect the increased distending pressure in the coronary arteries of C57BL/6N TAC mice.

As expected, no coronary atherosclerosis was observed in C57BL/6N apo E^−/−^ sham mice. In contrast, advanced lesions were observed in the left coronary artery of C57BL/6N apo E^−/−^ TAC mice (Figure 3, Table 2), indicating that increased coronary pressure and hypercholesterolemia caused by increased remnant lipoproteins interact to produce these coronary artery lesions. The degree of outward remodeling and the degree of media hypertrophy in C57BL/6N apo E^−/−^ TAC mice were similar compared to C57BL/6N TAC mice. The luminal area was decreased by 28.1% (*p* < 0.05) in C57BL/6N apo E^−/−^ TAC mice compared to C57BL/6N TAC mice. However, because of outward remodeling, the luminal area was 1.73-fold (*p* < 0.01) higher in C57BL/6N apo E^−/−^ TAC mice than in C57BL/6N apo E^−/−^ sham mice (Table 2). Taken together, C57BL/6N apo E^−/−^ TAC mice constitute a model of coronary atherosclerosis.

Area within the external elastic lamina (EEL), area within the internal elastic lamina (IEL), medial area, intimal area, luminal area, and percentage stenosis in the left coronary artery in the four experimental conditions.

### 3.3. Pressure Overload Induced by TAC Results in Markedly Increased Cardiac Hypertrophy and More Pronounced Heart Failure in C57BL/6N Apo E^−/−^ Mice in Comparison to C57BL/6N Mice

**Figure 4:**
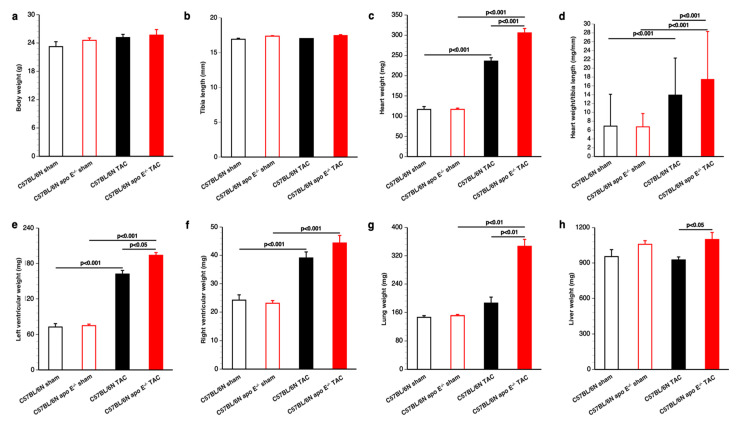
Cardiac hypertrophy and heart failure after TAC. Body weight (panel (**a**)), tibia length (panel (**b**)), heart weight (panel (**c**)), heart weight/tibia length (panel (**d**)), left ventricular weight (panel (**e**)), right ventricular weight (panel (**f**)), lung weight (panel (**g**)), and liver weight (panel (**h**)) in female C57BL/6N sham mice (n = 16), female C57BL/6N apo E^−/−^ sham mice (n = 9), female C57BL/6N TAC mice (n = 14), and female C57BL/6N apo E^−/−^ TAC mice (n = 11). Sham mice are represented by open bars whereas TAC mice are represented by closed bars. C57BL/6N mice are represented in black whereas C57BL/6N apo E^−/−^ mice are represented in red.

Body weight, tibia length, and organ and tissue weights of mice that underwent euthanasia 8 weeks after TAC or sham surgery are summarized in Figure 4. Neither body weight (Figure 4a) nor tibia length (Figure 4b) was significantly different between the four conditions. Total heart weight was increased by 2.02-fold (*p* < 0.001) in C57BL/6N TAC mice and by 2.62-fold (*p* < 0.001) in C57BL/6N apo E^−/−^ TAC mice compared to respective sham groups. Cardiac weight was 1.30-fold (*p* < 0.001) higher in C57BL/6N apo E^−/−^ TAC mice than in C57BL/6N TAC mice (Figure 4c). Similar differences were observed when heart weight was normalized to tibia length (Figure 4d). Left ventricular weight was 2.23-fold (*p* < 0.001) higher in C57BL/6N TAC mice (*p* < 0.001) and 2.59-fold (*p* < 0.001) higher in C57BL/6N apo E^−/−^ TAC mice than in respective sham groups (Figure 4e). Left ventricular weight was increased by 1.20-fold (*p* < 0.05) in C57BL/6N apo E^−/−^ TAC mice compared to C57BL/6N TAC mice (Figure 4e). Furthermore, right ventricular weight was increased by 1.61-fold (*p* < 0.001) in C57BL/6N TAC mice and by 1.92-fold (*p* < 0.001) in C57BL/6N apo E^−/−^ TAC mice compared to respective sham groups (Figure 4f). Wet lung weight was 1.27-fold (*p* < 0.001) higher in C57BL/6N TAC mice and 2.29-fold (*p* < 0.001) higher in C57BL/6N apo E^−/−^ TAC mice than in respective sham groups (Figure 4g). Wet lung weight was increased by 1.86-fold (*p* < 0001) in C57BL/6N apo E^−/−^ TAC mice compared to C57BL/6N TAC mice, indicating much more pronounced heart failure in the former (Figure 4g). No significant increase in liver weight was observed in TAC mice compared to sham mice. Taken together, pressure overload induced by TAC resulted in much more pronounced cardiac hypertrophy and heart failure in apo E deficient mice compared to wild-type mice.

### 3.4. Increased Myocardial Fibrosis and Capillary Rarefaction following TAC in Apo E Deficient Mice

**Figure 5:**
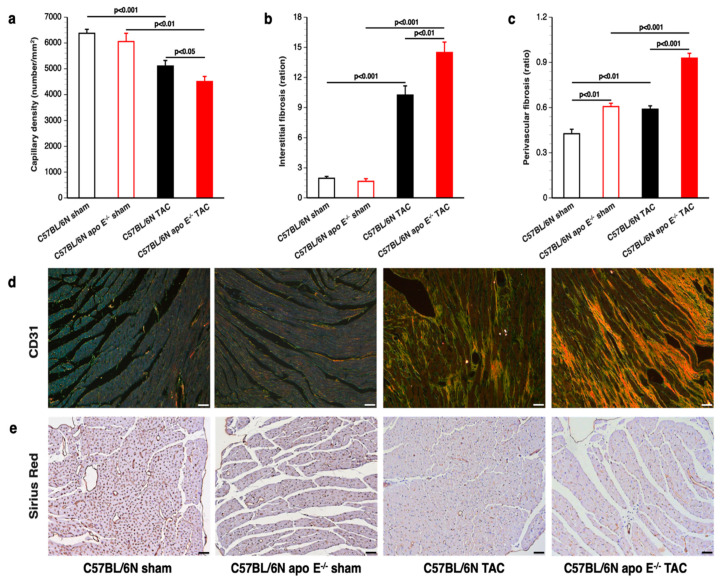
Histological analysis of pathological hypertrophy after TAC. Myocardial capillary density (panel (**a**)), interstitial fibrosis (panel (**b**)), and perivascular fibrosis (panel (**c**)) in female C57BL/6N sham mice, female C57BL/6N apo E^−/−^ sham mice, female C57BL/6N TAC mice, and female C57BL/6N apo E^−/−^ TAC mice. Representative photomicrographs show Sirius Red-stained interstitial collagen viewed under polarized light (panel (**d**)) and CD31-positive capillaries (panel (**e**)). The scale bar represents 50 µm.

Features of pathological hypertrophy are capillary rarefaction and myocardial fibrosis. Myocardial capillary density was reduced by 19.9% (*p* < 0.001) in C57BL/6N TAC mice and by 25.6% (*p* < 0.001) in C57BL/6N apo E^−/−^ TAC mice compared to respective sham groups (Figure 5a). Myocardial capillary density was 11.7% (*p* < 0.05) lower in C57BL/6N apo E^−/−^ TAC mice than in C57BL/6N TAC mice (Figure 5a). TAC induced pronounced interstitial myocardial fibrosis in both C57BL/6N TAC mice and in C57BL/6N apo E^−/−^ TAC mice. Myocardial fibrosis was increased by 1.41-fold (*p* < 0.01) in C57BL/6N apo E^−/−^ TAC mice compared to C57BL/6N TAC mice (Figure 5b). Perivascular fibrosis was 1.57-fold (*p* < 0.001) higher in C57BL/6N apo E^−/−^ TAC mice than in C57BL/6N TAC mice (Figure 5c). Representative photomicrographs showing Sirius Red-stained interstitial collagen viewed under polarized light are shown in Figure 5d. Figure 5e contains representative photomicrographs of CD31-positive vessels.

### 3.5. Deterioration of Cardiac Function Induced by TAC Is Significantly More Pronounced in Apo E Deficient Mice Compared to Wild-Type Mice

Diastolic dysfunction was observed in C57BL/6N apo E^−/−^ sham mice, as evidenced by a 13.1% (*p* < 0.05) decrease in the absolute value of the maximal rate of isovolumetric relaxation and a 1.12-fold (*p* < 0.05) increase in the time constant of isovolumetric relaxation (Table 3). Pressure overload induced by TAC in apo E deficient mice induced a pronounced (*p* < 0.05) increase in end-diastolic pressure compared to wild-type TAC mice (Table 4). Diastolic function was worse in C57BL/6N apo E^−/−^ TAC mice compared to C57BL/6N TAC mice, as evidenced by a 1.18-fold (*p* < 0.05) increase in the time constant of isovolumetric relaxation (Table 4).

### 3.6. Apo E Deficiency Induces Pronounced Oxidative Stress in Both Sham Mice and TAC Mice

**Figure 6:**
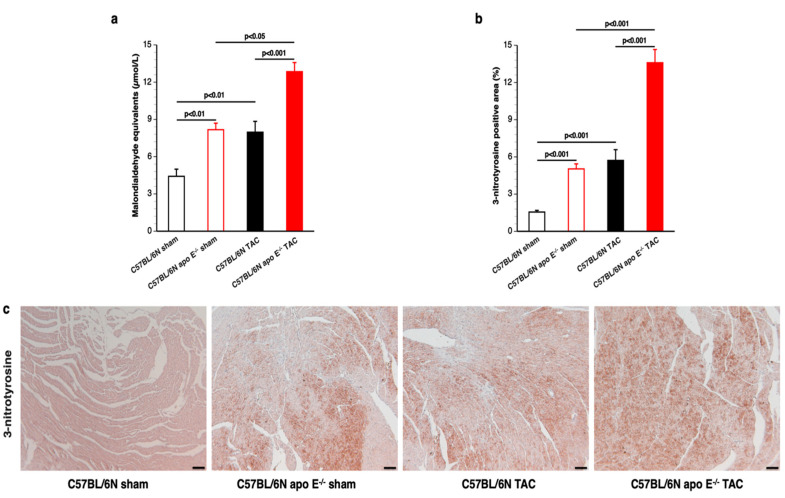
Quantification of oxidative stress in sham mice and in TAC mice. Sham mice and TAC mice are indicated by open bars and closed bars, respectively. C57BL/6N mice are represented in black whereas C57BL/6N apo E^−/−^ mice are represented in red. Plasma TBARS expressed as plasma malondialdehyde equivalents (panel (**a**)). Percentage of 3-nitrotyrosine-positive area in the myocardium (panel (**b**)). Quantifications in panel (**a**,**b**) were performed in 10 female C57BL/6N sham mice, 10 female C57BL/6N apo E^−/−^ sham mice, 21 female C57BL/6N TAC mice, and 31 female C57BL/6N apo E^−/−^ TAC mice. Representative photomicrographs showing myocardial sections stained for 3-nitrotyrosine (panel (**c**)). The scale bar represents 50 µm.

Lipid peroxidation was quantified by the measurement of Thiobarbituric Acid Reactive Substances (TBARS) in plasma. TBARS were increased by 1.85-fold (*p* < 0.01) in C57BL/6N apo E^−/−^ sham mice compared to C57BL/6N sham mice (Figure 6a). Pressure overload induced a prominent increase in TBARS levels. TBARS were 1.80-fold (*p* < 0.01) higher in C57BL/6N TAC mice and 1.57-fold (*p* < 0.05) higher in C57BL/6N apo E^−/−^ TAC mice compared to respective sham groups. The level of lipid peroxidation products was increased by 1.61-fold (*p* < 0.001) in C57BL/6N apo E^−/−^ TAC mice compared to C57BL/6N TAC mice (Figure 6a). The same pattern of differences was observed when the 3-nitrotyrosine positive area was quantified in the myocardium (Figure 6b). The effect of apo E deficiency on the 3-nitrotyrosine positive area was both observed in sham mice (3.24-fold increase; *p* < 0.001) and in TAC mice (2.38-fold increase; *p* < 0.001) (Figure 6b). Representative images of myocardial sections immunostained for 3-nitrotyrosine are shown in Figure 6c.

## 4. Discussion

The main findings of this study are that (1) increased coronary artery pressure induced by TAC on the one hand and increased remnant lipoproteins, on the other hand, interact to produce coronary atherosclerosis in apo E deficient mice; (2) pressure overload induced by TAC leads to much more pronounced cardiac hypertrophy and much more prominent features of pathological hypertrophy in apo E deficient mice compared to wild-type mice; (3) heart failure in C57BL/6N apo E^−/−^ TAC mice is much more marked than in wild-type mice as evidenced by the increased end-diastolic pressure and the remarkable augmentation of the lung weight; (4) increased oxidative stress is not only observed in TAC mice but also in C57BL/6N apo E^−/−^ sham mice, indicating that apo E deficiency is a model of increased oxidative stress.

Predilection sites for atherosclerosis in apo E^−/−^ mice as well as in low-density lipoprotein receptor (LDLr) deficient mice are the aortic root, the lesser curvature of the aortic arch, and the brachiocephalic artery [16,34,35,36]. However, mice are very resistant to coronary atherosclerosis [16]. This may be related to specific characteristics of the anatomy of coronary arteries in mice. Indeed, major anatomical differences exist between human coronary arteries and murine coronary arteries [37]. Similarly, as in other rodent species, the murine heart has no interventricular grooves, and the left and right coronary arteries become intramyocardial very shortly after their origin [37]. Thus, in contrast to humans, the proximal part of the coronary arteries has an intramyocardial course and not an epicardial course. Moreover, since there is no interventricular groove, there is no artery that is truly equivalent to the left anterior descending coronary artery in humans. Rather, the left main coronary arterial trunk divides into the left circumflex on the one hand and the obtuse marginal arteries on the other hand that supply the ventral and dorsal walls of the left ventricle [37]. In species with intramyocardial coronary arteries, the interventricular septum is mainly irrigated by a distinct artery, the septal artery [37]. A single major septal coronary artery in mice may arise either from a separate ostium from the right sinus of Valsalva or originate as a proximal branch of the right coronary artery or less frequently from the left coronary artery [37,38,39]. Occasionally two septal arteries are present [37,38]. When taken together, the absence of an interventricular groove is associated with an intramyocardial course of coronary arteries, the absence of a left anterior descending coronary artery, and the presence of one or two septal arteries.

Coronary atherosclerosis in humans develops in the epicardial coronary arteries and not in the intramyocardial arteries [18]. Therefore, it is not surprising that, in general, no coronary atherosclerosis is observed in the two most commonly used murine models of hypercholesterolemia [16,34,35,36]. One explanation for the absence of atherosclerosis in intramyocardial arteries is the lower or absent transmural pressure gradient [18]. Cardiac contraction compresses intramyocardial vessels and moves the plasma, pro-atherogenic lipoproteins, and infiltrating leukocytes away from the coronary artery wall [40]. Myocardial bridging is a congenital anomaly in which a segment of a coronary artery is surrounded by a myocardium, and the artery covered by the myocardium is called a tunneled artery. The tunneled segment is spared from atherosclerosis [41]. An alternative explanation for the absence of coronary atherosclerosis in intramyocardial vessels is the absence of vasa vasorum in the intramyocardial arteries [42]. The absence of adventitial vasa vasorum formation is also observed at the coronary segment covered by a myocardial bridge [43].

TAC in mice will increase coronary artery pressure and the distending pressure in the coronary artery. Changes in coronary pressure affect coronary dimensions [44]. The increased distending pressure resulted in an increase in luminal area in both C57BL/6N TAC mice and C57BL/6N apo E^−/−^ TAC mice. Moreover, increased pressure is the cause of media hypertrophy [45]. The structural changes of the artery and/or changes in transmural pressure gradient following TAC potentiate the development of atherosclerosis in C57BL/6N apo E^−/−^ TAC mice. In contrast to the presence of extensive lesions in female C57BL/6N apo E^−/−^ TAC mice, no coronary atherosclerosis at all has previously been observed in female C57BL/6J LDLr^−/−^ TAC mice on standard chow [21] or on a 0.2% cholesterol 10% coconut oil diet [22]. This cannot be attributed to differences in plasma cholesterol levels since total plasma cholesterol levels in C57BL/6J LDLr^−/−^ mice on a 0.2% cholesterol 10% coconut oil are higher than in C57BL/6N apo E^−/−^ mice fed standard chow. Therefore, differences in the development of coronary atherosclerosis must be related to differences in the nature of pro-atherogenic lipoproteins between apo E deficient mice and LDLr deficient mice, properties of apo E independent of its effects on lipid levels, or differences in the genetic background (C57BL/6N versus C57BL/6J). Apo E deficient mice are characterized by increased concentration of chylomicron remnants and VLDL remnants, which are contained in β-VLDL particles and intermediate density lipoprotein (IDL) particles [46,47,48]. LDL receptor-deficient mice have a much more selective increase in LDL [49,50]. Furthermore, LDL receptor-deficient mice are characterized by a pronounced increase in apo B-100 and a modest elevation of apoB-48, whereas apo E deficient mice have a striking increase in apo B-48 but not of apoB-100 [51]. Therefore, the pro-atherogenic lipoproteins in apo E deficient mice are apo B-48-containing remnants, whereas the pro-atherogenic lipoproteins in LDL receptor-deficient mice are apo B-100-containing LDL. These differences may be relevant to the observed difference in coronary atherosclerosis. Remnant lipoprotein cholesterol is a predictor of incident coronary heart disease in humans [24,25,52].

Apart from its effects on lipoprotein metabolism and lipoprotein levels, lipoprotein-independent functions have also been attributed to apo E [53]. These functions include effects on immune response, inflammation and oxidation, and on smooth muscle proliferation and migration. Although these lipoprotein-independent effects may theoretically contribute to the development of atherosclerosis in apo E deficient mice, this hypothesis cannot be corroborated by direct experimental evidence. In contrast, multiple studies have demonstrated that C57BL/6J and C57BL/6N mice present various phenotypic differences with relevance to cardiovascular research [54,55]. C57BL/6J mice are characterized by a spontaneous mutation in the nicotinamide nucleotide transhydrogenase gene [56,57]. This enzyme catalyzes the reduction of NADP^+^ at the price of oxidation of NADH oxidation and proton re-entry into the mitochondrial matrix. Functional loss of this enzyme due to this spontaneous mutation in C57BL/6J mice results in mitochondrial redox abnormalities that result in a poor capacity to sustain NADP and glutathione in their reduced states. This mutation may be relevant both for the development of atherosclerosis [54,58] and for the development of cardiac dysfunction. However, since apo E deficient mice are on the C57BL/6N background and LDLr deficient mice are on the C57BL/6J background, this mutation does not explain the presence of coronary atherosclerosis in C57BL/6N apo E^−/−^ TAC mice and the absence of coronary atherosclerosis in C57BL/6J LDLr^−/−^ TAC mice. However, since the mutation in the nicotinamide nucleotide transhydrogenase gene is only one of many genetic factors that may affect cardiovascular phenotypic differences between C57BL/6J and C57BL/6N mice [55,57], we cannot exclude that the genetic background places a role in the coronary phenotype of both models.

TAC leads to much more marked cardiac hypertrophy and much more prominent features of pathological hypertrophy in apo E deficient mice compared to wild-type C57BL/6N mice. Notwithstanding the presence of coronary atherosclerosis, the luminal area was increased in C57BL/6N apo E^−/−^ TAC mice compared to C57BL/6N apo E^−/−^ sham mice. Therefore, TAC in C57BL/6N apo E^−/−^ mice remains a model of non-ischemic cardiomyopathy. The direct impact of hypercholesterolemia on cardiac hypertrophy and cardiac function has previously been demonstrated both in the absence of pressure overload [59,60,61] and in the presence of pressure overload [21,22]. Hypercholesterolemia also potentiates cardiac hypertrophy and adverse remodeling following myocardial infarction [31,62] and in the setting of diabetic cardiomyopathy [63]. Oxidative stress and nitro-oxidative stress may play a key role in the effects of hypercholesterolemia on cardiac structure and function [64,65,66]. Hypercholesterolemia also induces hyperactive mammalian target of rapamycin (mTOR) signaling in the heart, whereby protein synthesis and cell growth are promoted by the mTOR complex 1 (mTORC1) [22,67,68].

Pathological hypertrophy and cardiac dysfunction in C57BL/6N apo E^−/−^ TAC mice resulted in pronounced heart failure, as evidenced by the very marked increase in the wet lung weight, which is much more prominent compared to other models of TAC-induced heart failure [21,22]. This raises the hypothesis that high concentrations of remnant lipoproteins may be more detrimental to the myocardium than high concentrations of LDL. Alternatively, the more pronounced effects of hypercholesteremia on the development of heart failure in apo E deficient mice may be related to the impact of apo E deficiency per se on inflammation and on oxidation. Apo E modulates several dimensions of the inflammatory response [53,69,70,71] and also exerts anti-oxidant effects in vivo [53]. This may clarify why oxidative stress was elevated in apo E deficient mice even in the absence of TAC. Since inflammation [72] and oxidation [66] contribute to the development of heart failure, this may explain why apo E deficiency per se may be a potent potentiator of pressure-induced heart failure. Increased oxidative stress may also underlie diastolic dysfunction in C57BL/6N apo E^−/−^ sham mice [73].

TAC in C57BL/6N apo E deficient mice resulted in higher mortality than in C57BL/6N mice. This may partially reflect a higher incidence of death caused by acute heart failure induced by the hemodynamic effects of pressure overload but may also reflect an increased incidence of lethal arrhythmias. The pro-arrhythmic effects of hypercholesterolemia may be mediated through direct interactions between cholesterol and the membrane proteins, through alterations of membrane fluidity, and/or the impact of hypercholesterolemia on lipid rafts [74,75].

The current study highlights the importance of interaction between different risk factors in the development of coronary atherosclerosis and potentially also in the development of heart failure. In clinical medicine, the effects of two causes are frequently not additive, but the impact of one causal variable on an outcome may be dependent on the state of a second causal variable. In particular, interactions between genotype and environmental factors should be considered, and the impact of genetic risk factors may therefore be conditional on specific environmental exposures. Murine atherosclerosis models are generally hampered by the absence of coronary atherosclerosis. The model investigated in this study offers perspectives for evaluating the effect of pharmacological interventions in a robust model of coronary atherosclerosis in mice. The C57BL/6N apo E^−/−^ TAC mice also constitute a model of severe oxidative stress and heart failure and may be used to evaluate novel therapeutic heart failure strategies in mice.

In conclusion, the interaction between increased remnant lipoproteins and increased coronary pressure induced by TAC produces advanced coronary atherosclerosis in apo E deficient mice. Pressure overload-induced cardiomyopathy in apo E deficient mice is characterized by a very severe phenotype with prominent features of pathological remodeling and very pronounced heart failure. Apo E deficient mice are a model of increased oxidative stress, which may explain diastolic dysfunction in C57BL/6N apo E^−/−^ sham mice.

## Figures and Tables

**Table 1:** Total, non-HDL, and HDL plasma cholesterol levels in female C57BL/6N and female C57BL/6N apo E^−/−^ mice.

	C57BL/6N(*n* = 5)	C57BL/6N Apo E^−/−^(*n* = 10)
Total cholesterol	68.3 ± 2.4	296 ± 4 ****
Non-HDL cholesterol	9.39 ± 1.50	274 ± 4 ****
HDL cholesterol	58.9 ± 1.2	22.5 ± 1.3 ****

Data are expressed as means ± SEM. Lipoproteins were separated by gel filtration. ****: *p* < 0.0001 versus C57BL/6.

**Table 2:** Coronary atherosclerosis in female C57BL/6N TAC apo E^−/−^ mice.

	C57BL/6N Sham (n = 9)	C57BL/6N Apo E^−/−^ Sham (n = 12)	C57BL/6N TAC (n = 16)	C57BL/6N Apo E^−/−^TAC (n = 24)
EEL area (µm^2^)	30,000 ± 1800	29,300 ± 2700	65,600 ± 4900 ^§§§^	67,800 ± 8100 ^!!!^
IEL area (µm^2^)	17,800 ± 400	18,500 ± 1700	44,400 ± 3700 ^§§§^	43,200 ± 5400 ^!!^
Media area (µm^2^)	12,200 ± 1600	10,800 ± 1100	21,200 ± 2100 ^§§^	24,700 ± 2800 ^!!!^
Intima area (µm^2^)	0.00 ± 0.01	0.00 ± 0.01	0.00 ± 0.01	11,300 ±3500 ^!!!^***
Luminal area (µm^2^)	17,800 ± 400	18,500 ± 1700	44,400 ± 3700 ^§§§^	31,900 ± 3200 ^!!^*
Stenosis (%)	0.00 ± 0.01	0.00 ± 0.01	0.00 ± 0.01	18.9 ± 4.1 ^!!!^***

Stenosis is defined as the ratio of intimal area divided by IEL area and is expressed as percentage. ^§§^: *p* < 0.01; ^§§§^: *p* < 001 versus C57BL/6N sham. ^!!^: *p* < 0.01; ^!!!^: *p* < 0.001 versus C57BL/6N apo E^−/−^ sham. *: *p* < 0.05 versus C57BL/6N TAC. ***: *p* < 0.001 versus C57BL/6N TAC.

**Table 3:** Hemodynamic parameters in the left ventricle and in the aorta 8 weeks after sham operation in female C57BL/6N mice and female C57BL/6N apo E^−/−^ mice. Sham operation was performed at the age of 14 weeks. Data are expressed as means ± SEM. °: *p* < 0.05 versus C57BL/6N sham.

	C57BL/6N Sham (n = 19)	C57BL/6N Apo E^−/−^ Sham (n = 8)
**LEFT VENTRICLE**		
Peak systolic pressure (mm Hg)	101 ± 2	96.1 ± 1.3
End-diastolic pressure (mm Hg)	0.677 ± 0.475	1.96 ± 0.46
dP/dt_max_ (mm Hg/ms)	12.1 ± 0.4	10.5 ± 0.7
dP/dt_min_ (mm Hg/ms)	−9.91 ± 0.35	−8.61 ± 0.42°
Tau (ms)	4.42 ± 0.13	4.94 ± 0.15°
Heart rate (bpm)	609 ± 13	599 ± 12
**AORTA**		
Mean pressure (mm Hg)	81.6 ± 1.7	79.5 ± 2.0
Systolic pressure (mm Hg)	99.4 ± 1.7	95.6 ± 1.8
Diastolic pressure (mm Hg)	64.4 ± 2.6	64.1 ± 4.7

**Table 4:** Hemodynamic parameters in the left ventricle and in the aorta 8 weeks after TAC operation in female C57BL/6N mice and female C57BL/6N apo E^−/−^ mice. Sham operation was performed at the age of 14 weeks. Data are expressed as means ± SEM. *: *p* < 0.05 versus C57BL/6N TAC.

	C57BL/6N TAC (n = 18)	C57BL/6N Apo E^−/−^ TAC (n = 21)
**LEFT VENTRICLE**		
Peak systolic pressure (mm Hg)	173 ± 9	183 ± 8
End-diastolic pressure (mm Hg)	2.29 ± 0.90	6.32 ± 1.04 *
dP/dt_max_ (mm Hg/ms)	11.9 ± 0.8	11.9 ± 0.7
dP/dt_min_ (mm Hg/ms)	−11.5 ± 0.6	−11.3 ± 0.7
Tau (ms)	5.10 ± 0.23	6.00 ± 0.33 *
Heart rate (bpm)	631 ± 11	601 ± 12
**AORTA**		
Mean pressure (mm Hg)	104 ± 6	109 ± 4
Systolic pressure (mm Hg)	172 ± 11	183 ± 8
Diastolic pressure (mm Hg)	61.8 ± 5.5	65.1 ± 5.5

## Data Availability

The data presented in this study are available on motivated request directed to the corresponding author.
